# Phylogenetic Study of Polyketide Synthases and Nonribosomal Peptide Synthetases Involved in the Biosynthesis of Mycotoxins

**DOI:** 10.3390/toxins5040717

**Published:** 2013-04-19

**Authors:** Antonia Gallo, Massimo Ferrara, Giancarlo Perrone

**Affiliations:** Institute of Sciences of Food Production (ISPA), National Research Council (CNR), Bari, Italy; E-Mails: massimo.ferrara@ispa.cnr.it (M.F.); giancarlo.perrone@ispa.cnr.it (G.P.)

**Keywords:** polyketide synthase, non-ribosomal peptide synthetase, phylogenies, β-ketosynthase, acyl-transferase, adenylation, condensation, secondary metabolites

## Abstract

Polyketide synthase (PKSs) and nonribosomal peptide synthetase (NRPSs) are large multimodular enzymes involved in biosynthesis of polyketide and peptide toxins produced by fungi. Furthermore, hybrid enzymes, in which a reducing PKS region is fused to a single NRPS module, are also responsible of the synthesis of peptide-polyketide metabolites in fungi. The genes encoding for PKSs and NRPSs have been exposed to complex evolutionary mechanisms, which have determined the great number and diversity of metabolites. In this study, we considered the most important polyketide and peptide mycotoxins and, for the first time, a phylogenetic analysis of both PKSs and NRPSs involved in their biosynthesis was assessed using two domains for each enzyme: β-ketosynthase (KS) and acyl-transferase (AT) for PKSs; adenylation (A) and condensation (C) for NRPSs. The analysis of both KS and AT domains confirmed the differentiation of the three classes of highly, partially and non-reducing PKSs. Hybrid PKS-NRPSs involved in mycotoxins biosynthesis grouped together in the phylogenetic trees of all the domains analyzed. For most mycotoxins, the corresponding biosynthetic enzymes from distinct fungal species grouped together, except for PKS and NRPS involved in ochratoxin A biosynthesis, for which an unlike process of evolution could be hypothesized in different species.

## 1. Introduction

Polyketide synthase (PKSs) and non-ribosomal peptide synthetase (NRPSs) are large multimodular enzymes involved in biosynthesis of polyketide and peptide secondary metabolites produced by microorganisms, such as bacteria and fungi. In addition to natural products that display a broad range of useful activities for pharmaceutical properties, including antibiotics (penicillins) and possible anticancer (gliotoxin) and cholesterol lowering (lovastatin/compactin) agents, a consistent number of fungal derived PK and NRP metabolites present negative mycotoxic properties, such as aflatoxins, ochratoxin A (OTA), fumonisin and patulin, among the most important. These enzymes employ complimentary strategies to sequentially construct a diverse array of compounds from relatively simple carboxylic and amino acid building blocks [1,2]. In PKS assembly lines, the monomers are acyl-CoA thioesters (acetyl-CoA, malonyl-CoA, methylmalonyl-CoA), derived from the pool of primary metabolites in microbial producer cells. The monomers for NRPS assembly are proteinogenic and nonproteinogenic amino acids and other carboxylic acids [3]. Polyketide synthases can be categorized into three different types (I, II and III), which have the same enzymatic functions, but difference in quaternary structure and chain initiation and termination. Type I PKSs are large enzymes comprised of multiple functional domains acting only one time during the biosynthesis; type II are a complex of several single module proteins with separated enzymatic activities, acting iteratively to produce a polyketide; type III are characterized by a single active site enzyme that acts repeatedly to form the final product; they function as homodimers, do not include an acyl-carrier protein (ACP) domain and are typically associated to plants, even though there are examples of type III PKSs also in bacteria and fungi. While bacteria possess both non-iterative type I PKS containing individual modules with all necessary domains for every single elongation step and iterative type II PKS, in fungi, PKSs are mainly iterative type I enzymes, because they have a modular organization like type I, but the catalytic site of each enzymatic domain acts repeatedly to elongate the polyketide backbone. Polyketide synthase modules contain three main domains: β-ketosynthase (KS), acyl-transferase (AT) and ACP domains. The KS domain attaches a malonyl-CoA extender unit to an acetyl-CoA starter molecule; the AT domain supports the loading of correct substrate onto the enzyme; the ACP domain is responsible for facilitating the movement of substrates and products between the different active sites within the enzyme. The three domains are used in each catalytic cycle, resulting in the elongation of the polyketide chain by a single ketide unit. Optionally, each elongation step can be followed by further β-keto processing steps, mediated by β-ketoreductase (KR), dehydratase (DH) and trans-acting enoyl (ER) domains [4,5]. According to the absence or the presence of these reducing functions, fungal PKSs can be divided into non-reducing (NR) and highly reducing (HR) PKS, respectively; whereas the presence of KR or KR and DH domains, but not ER domains, results in the intermediate class of partially reducing (PR) PKS [6]. However, in some cases, HR-PKSs can also lack a conserved ER domain, harboring instead a region without known function and, apparently, utilizing a freestanding ER *in trans*. Furthermore, optionally, methyltransferase domain (MeT) assists methylation of the polyketide carbon backbone during chain formation; the thioesterase (TE) domain promotes the hydrolysis of the thioester and releases the carbon chain from the enzyme using water, whereas Claisen cyclase (CYC) domain promotes the product release using an intramolecular reaction. In addition, PKSs can undergo post-assembly line modifications by *O*-methyl transferase, glycosylase and other enzymes [7]. Non-reducing PKSs lack reducing functions, but instead contain at the N-terminus the “starter unit-ACP transacylase” (SAT) domain, responsible for selection and loading of a starter unit, and the product template (PT) domain, usually located after AT domain, for the controlled folding of the PK backbone [8]. Unlike NR-PKS, HR-PKS analyzed to date do not contain SAT domains; in PR-PKS there are no SAT, PT or TE domains.

Fungal NRPSs are multifunctional enzymes composed of enzymatic modules used to elongate the amino acid chain. In NRPS, according to the collinearity rule, the number and the order of the modules represents the number and the order of amino acids in the final product [4,9], but there are exceptions, as some modules can be used repeatedly by the growing peptide chain, while others are bypassed [10]. A typical NRPS module consists minimally of an adenylation (A) domain, responsible for recognition and activation of its related amino acid or hydroxyl acid, the thiolation or peptidyl-carrier protein (T or PCP) domain, which acts as a swinging arm carrying a phosphopantetheinyl at a conserved serine residue, that transports substrates between the active sites of the domains, and the condensation (C) domain, which catalyzes the formation of the peptide bond (C-N) between the elongated chain and the activated amino acid. Adenylation domains are specific for one particular amino acid; thus, the order of adenylation domains within the synthetase is co-linear with the order of amino acids in the natural product. These core domains are often supported by tailoring domains, such as the epimerization (E) domain, which changes an l-amino acid into a d-amino acid, as well as the dual/epimerization domains (E/C), which are responsible for both epimerization and condensation. Other optional domains are the reductase (R) domain, important for reducing of the final peptide, the methylation (MT) domain, which assists the N-methylation of the amide nitrogen, the cyclization (Cy) domain, for the formation of heterocyclic rings, and the oxidation (Ox) domain, usually located either downstream of the PCP domain or in the C-terminus of the A domain, which catalyzes the formation of an aromatic thiazol through oxidation of a thiazoline ring. Thioesterase domains (TE) are usually located in the final NRPS module and help the release of the final peptide from the enzyme through cyclization or hydrolysis [11,12,13,14,15]. The final peptide product may be decorated with lipid and/or carbohydrate moieties, so that it can exhibit a diverse array of structures and biological effects [16]. As well as linear, the assembly lines of NRPSs can also be iterative and non-linear. Iterative NRPSs use their modules or domains repeatedly during the assembly of a product, whereas for NRPSs, whose architecture is unusual, the domain interactions are more complex and their possible product cannot be predicted and are named non-linear NRPSs. Most fungal NRPSs described so far are of the linear or iterative type [16,17]

Hybrid peptide-polyketide metabolites, in which polyketides are fused to an amino acid by an amide bond, may be synthesized individually by NRPS and PKS without any direct functional hybridization, but eventually coupled in a hybrid final product. Alternatively, PKS is fused to a single NRPS module, which is sometimes truncated, to form a hybrid enzyme, so that, after a defined number of elongation steps, the polyketide intermediate is transferred to the NRPS module to be linked to the activated amino acid through a peptide bond [18]. Fungal PKS-NRPS hybrids have a reducing PKS region, with the characteristic domains, flanked by the C, A and PCP domains of a typical NRPS module. Furthermore, the hybrid systems could exhibit an additional C-terminal domain that might function as either a thioester reductase (R) or cyclase domain.

The major classes of mycotoxins derived from polyketide metabolism are among the most significant in agriculture and food industry: aflatoxins, fumonisins, ochratoxins and zearalenone. The biosynthetic pathway of some of them needs the enzymatic activity of a NRPS. Other PK-derived mycotoxins are emerging as important food and feed contaminants, such as fusaproliferin, beauvericin, enniatins and moniliformin [19]. 

The genes encoding for PKSs and NRPSs are among the largest found in microbial genomes. Usually, these genes together with tailoring and regulatory genes tend to exist in biosynthetic clusters, that is, they are located at the same locus in the genome and are coexpressed [20]. Sequence analyses of fungal genomes have uncovered a high number of putative biosynthetic gene clusters, including PKS, NRPS and/or PKS-NRPS hybrid genes. During the last decade rapid development of bioinformatic tools, as well as improved sequencing and annotation of microbial genomes led to discovery of novel bioactive compounds synthesized by NRPS and PKS by means of genome mining [21,22], but given the number of putative secondary metabolites genes that have been found, many corresponding natural products have yet to be discovered. 

The evolutionary mechanism by which these clusters are created and maintained are unclear. Natural selection could have acted by near simultaneous relocations of genes previously distributed in the genome [23], by chromatin remodeling based on co-regulation of expression, by linkage of interacting genes or by horizontal gene transfer (HGT) [24,25]. Other arguments concern the location of biosynthetic genes in genome regions that are particularly likely to recombine, such as the telomere ends of the chromosome [26], and the close proximity to mobile genetic elements [27]. Phylogenetic analysis could be of help for the understanding of the evolutionary mechanisms at the basis of the great number and diversity of these enzymes present in fungal genomes. Furthermore, phylogenomics provides a useful approach to deduce gene function and novel pathways and compounds based on phylogenetic relationship as opposed to sequence similarities [28]. Because of the size and complexity of these multidomain enzymes, the evolutionary histories based on the whole sequences of PKSs and NRPSs are largely uninformative, while phylogenies of their most highly conserved domains, reveal highly supported clustering patterns. Phylogenetic analyses suggested different mechanisms of evolution for NRPSs and PKSs, like gene duplication, divergence and differential gene loss [29] and also the origin of fungal 6-MSAS-type PKSs and hybrid NRPS/PKS enzymes from bacterial ancestors through ancient HGT events [30] or the clear distinction between type I and II PKSs [31]. From the study of Bushley and Turgeon [32] mono- and bi-modular NRPSs resulted in having a more ancient origin and more conserved domain architecture than most multimodular NRPSs, which are restricted to fungi, show less stable domain architectures and biosynthesize metabolites with more niche-specific functions. Domains phylogeny has also given indication on biochemical function, substrate specificity and structure motifs, as the grouping of PKSs in reducing and non-reducing clades [29]. Ketosynthase analyses in bacteria have also been used to predict pathway association and, in some cases, the secondary metabolic products of those pathway [33]. In this work, we present the results of phylogenetic analyses of KS and AT domains of PKSs and A and C domains of NRPSs involved in the biosynthesis of mycotoxins. The aim of this phylogenetic study was to identify the domain lineages associated to the secondary metabolite produced and to biosynthetic mechanisms and to assess the diversity of fungal mycotoxin genes for a better clarification of phylogenetic origin of these fundamental enzymes.

## 2. Results and Discussion

Recently, various phylogenetic analyses of fungal PKSs and NRPSs have been performed [29,32,34,35,36]. Most of these studies presented evolutionary relationships, taxonomic distribution and levels of conservation of these multimodular enzymes, focusing on only one of the two typology of enzymes and usually considering the genealogy of one of their characteristic domains. In our study, we have considered the most important polyketide and peptide mycotoxins, and this is the first paper in which phylogenetic analysis of PKSs, NRPSs and hybrid PKS-NRPSs involved in mycotoxins biosynthesis was assessed using two different conserved domains for each enzyme: KS and AT domains for PKSs and A and C domains for NRPSs. The enzymes analyzed in this study are listed in Table 1.

**Table 1:** Putative and known polyketide synthase (PKSs), nonribosomal peptide synthetase (NRPSs) and hybrid PKS-NRPSs analyzed in this study and identified by their accession numbers.

Mycotoxin	Code	Fungal species	PKS	NRPS	Hybrid PKS-NRPS
AFLATOXIN	*PksA*	*Aspergillus nomius*	Q5VD79		
AFLATOXIN	*PksA*	*Aspergillus flavus*	AAS89999		
AFLATOXIN	*PksL1*	*Aspergillus oryzae*	BAC45240		
AFLATOXIN	*PksL1*	*Aspergillus sojae*	AAU08792		
AFLATOXIN	*PksL1*	*Aspergillus parasiticus*	AAC41675		
AF-TOXIN	*AFT9-1*	*Alternaria alternata*	BAD97694		
ALTERNARIOL	*PksJ*	*Alternaria alternata*	AFN68301		
ALTERNARIOL	*PksH*	*Alternaria alternata*	AFN68299		
BEAUVERICIN	*BEAS*	*Beauveria bassiana*		ACI30655	
BEAUVERICIN	*putative*	*Fusarium oxysporum*		EGU75688	
BIKAVERIN	*Pks4*	*Gibberella fujikuroi*	CAB92399		
CERCOSPORIN		*Cercospora nicotianae*	AAT69682		
CERCOSPORIN	*CTB1*	*Cercospora coffeicola*	ADO14690		
CITRININ	*PksCT*	*Monascus purpureus*	AAY33862		
CITRININ		*Trichophyton tonsurans*	EGD97507		
CITRININ		*Coccidioides immitis*	EJB11047		
CITRININ	*putative*	*Aspergillus fumigatus*	XP_746971		
CITRININ	*putative*	*Neosartorya fischeri*	EAW17018		
CYCLOPIAZONIC ACID	*CpaA*	*Aspergillus flavus*			BAI43678
CYCLOPIAZONIC ACID	*CpaA*	*Aspergillus oryzae*			BAG82673
CHETOGLOBOSIN A	*CheA*	*Penicillium expansum*			CAO91861
CYTOCHALASIN E	*CcsA*	*Aspergillus clavatus*			XM_001270542
DOTHISTROMIN	*PksA*	*Mycosphaerella pini*	AAZ95017		
ENNIATIN	*Esyn1*	*Fusarium equiseti*		CAA79245	
ENNIATIN		*Fusarium verticillioides*		FVEG_09993	
EQUISETIN	*EqiS*	*Fusarium heterosporum*			Q5SBL2
ERGOT ALKALOIDS	*CPPS1*	*Claviceps purpurea*		CAB39315	
ERGOT ALKALOIDS	*CPPS2*	*Claviceps purpurea*		AJ439610	
ERGOT ALKALOIDS	*CPPS3*	*Claviceps purpurea*		AJ884677	
ERGOT ALKALOIDS	*CPPS4*	*Claviceps purpurea*		AJ884678	
FUMONISIN	*FUM1*	*Fusarium oxysporum*	ACB12550		
FUMONISIN	*FUM1*	*Gibberella moniliformis*	AF155773		
FUMONISIN	*FUM1-like*	*Aspergillus niger*	CAK43811		
FUMONISIN	*putative*	*Cochliobolus heterostrophus*	AAR90266		
FUMONISIN	*FUM10*	*Fusarium oxysporum*		ACB12556	
FUMONISIN	*FUM10- like*	*Aspergillus niger*		XP001389107	
FUMONISIN	*FUM14*	*Fusarium oxysporum*		ACB12559	
FUMONISIN	*FUM14*	*Fusarium verticillioides*		AAN74817	
FUMONISIN	*FUM14-like*	*Aspergillus niger*		XP_001389112	
FUMONISIN	*putative*	*Trichoderma atroviride*		EHK44571	
FUSARIN C	*FusA*	*Fusarium fujikuroi*			AFP73394
FUSARIN C	*FusS*	*Gibberella moniliformis*			AAT28740
FUSARIN C	*PKS10*	*Fusarium pseudograminearum*			EKJ71911
GLIOTOXIN	*GliP*	*Aspergillus fumigatus*		AAW03307	
GLIOTOXIN	*GliP*	*Neosartorya fischeri*		XP_001258083	
GLIOTOXIN	*GliP-like*	*Aspergillus flavus*		XP_002380016	
GLIOTOXIN	*GliP-like*	*Trichoderma virens*		EHK22005	
GLIOTOXIN	*putative*	*Zymoseptoria tritici*		XP_003854720	
HC TOXIN	*HTS1*	*Cochliobolus carbonum*		AAA33023	
HC TOXIN		*Penicillium digitatum*		EKV17246	
LOVASTATIN	*LovF*	*Aspergillus terreus*	AAD34559		
LOVASTATIN	*LovB*	*Aspergillus terreus*	Q9Y8A5		
LOVASTATIN		*Magnaporthe oryzae*	EHA58179		
LOVASTATIN	*LovB-like*	*Aspergillus fumigatus*	XP_751268		
LOVASTATIN	*putative*	*Neosartorya fischeri*	XP_001262312		
LOVASTATIN	*putative*	*Fusarium fujikuroi*	CAC44633		
LOVASTATIN	*putative*	*Aspergillus flavus*	XP_002385159		
LOVASTATIN	*putative*	*Aspergillus clavatus*	XP_001270376		
6-METHYL SALICILIC AC	*AtX*	*Aspergillus terreus*	BAA20102.2		
6-METHYL SALICILIC AC	*MsaS*	*Aspergillus clavatus*	XP_001273093		
6-METHYL SALICILIC AC	*6-MSAS*	*Penicillium griseofulvum*	P22367		
NAPHTHOPYRONE	*At4*	*Aspergillus terreus*	BAB88689		
NAPHTHOPYRONE	*YWA1*	*Arthroderma otae*	XP_002842704		
NAPHTHOPYRONE	*PksP/Alb1*	*Aspergillus fumigatus*	AAC39471		
NAPHTHOPYRONE	*WA*	*Aspergillus nidulans*	Q03149		
OCHRATOXIN A	*otapksPN/otanpsPN*	*Penicillium nordicum*	AAP33839	AAS98174	
OCHRATOXIN A		*Aspergillus ochraceus*	AAP32477/AAT92023		
OCHRATOXIN A		*Aspergillus niger*	XP_001397313	CAK42678	
OCHRATOXIN A		*Aspergillus carbonarius*	PI73482 *	PI 132610*	
OCHRATOXIN A		*Aspergillus carbonarius*	CAQ16344		
OCHRATOXIN A	*Aoks1*	*Aspergillus westerdijkiae*	AY583209		
OCHRATOXIN A	*putative*	*Penicillium verrucosum*		AEN14493	
OCHRATOXIN A	*putative*	*Aspergillus fumigatus*		XP748589	
OCHRATOXIN A	*putative*	*Aspergillus flavus*		XP_002375804	
OCHRATOXIN A	*putative*	*Neurospora crassa*		XP955820	
OCHRATOXIN A	*putative*	*Gibberella zeae*		XP390793	
PATULIN		*Penicillium expansum*	DQ084387		
PENICILLIN		*Penicillium chrysogenum*		P26046	
PENICILLIN	*AcvA*	*Aspergillus nidulans*		CBF84349	
PM-TOXIN	*PKS1*	*Mycosphaerella zeae-maydis*	AY495642		
PSEUROTIN A	*PsoA*	*Aspergillus fumigatus*			ABS87601
SIDEROPHORE	*SidC*	*Aspergillus fumigatus*		EAL91050	
SIDEROPHORE	*SidC*	*Aspergillus nidulans*		CBF89140	
SIRODESMIN	*SirP*	*Leptospheria maculans*		AAS92545	
STERIGMATOCYSTIN	*AflC*	*Aspergillus ochraceoroseus*	ACH72912		
STERIGMATOCYSTIN	*PksST*	*Aspergillus nidulans*	AAA81586		
T-TOXIN	*PKS1*	*Cochliobolus heterostrophus*	AAB08104		
T-TOXIN	*PKS2*	*Cochliobolus heterostrophus*	AY495643		
ZEARALENON	*PKS13*	*Gibberella zeae*	ABB90282		
ZEARALENON	*PKS4*	*Gibberella zeae*	ABB90283		

* http://jgi.doe.gov/carbonarius/

### 2.1. Analyses of KS and AT Domains of PKSs

**Figure 1:**
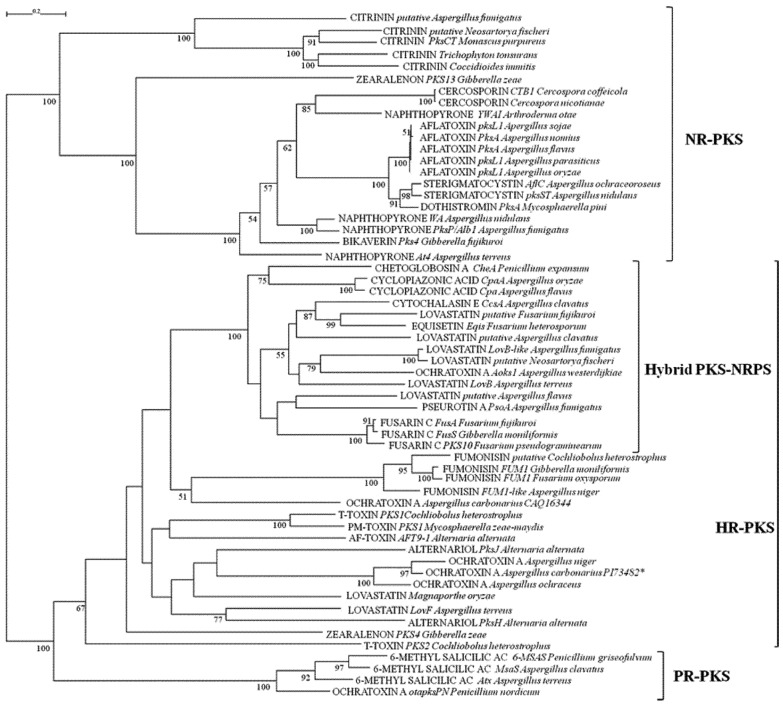
ML phylogenetic tree of β-ketosynthase (KS) domains from PKSs and hybrid PKS-NRPS based on RAxML algorithm. Numbers at nodes indicate bootstrap support, reported when ≥50% for each clade performed with 1,000 replications. NR: non-reducing; HR: highly reducing; PR: partially reducing.

**Figure 2:**
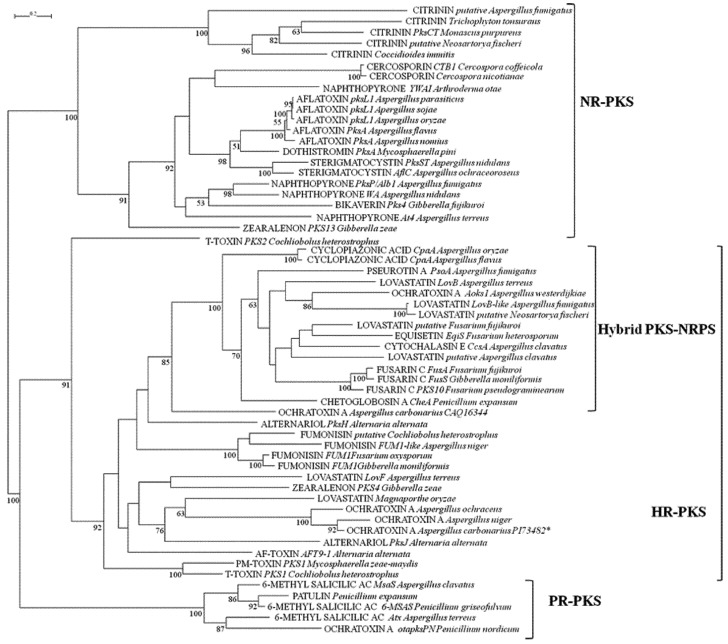
ML phylogenetic tree of acyl-transferase (AT) domains from PKSs and hybrid PKS-NRPS based on RAxML algorithm. Numbers at nodes indicate bootstrap support, reported when ≥50% for each clade performed with 1,000 replications. NR: non-reducing; HR: highly reducing; PR: partially reducing.

The KS and AT domains are the most conservative domains of PKSs. The results of both their phylogenetic analyses, as represented in Figure 1, Figure 2, coincide, allowing a primary classiﬁcation of PKSs into the same three clades, based on the differentiation in NR, PR and HR-PKSs. This latter clade also comprised PKS-NRPS hybrid synthases, which showed strong similarities in the arrangement of domains and fell into one distinctive subclade in both KS and AT phylogenetic analyses. The resolution of NR-PKSs involved in the biosynthesis of different mycotoxins into a single clade with a high bootstrap value (100%) supports the hypothesis that they share a common ancestor. In our analysis, NR-PKS group resulted also separated into two subclades. The first one consisted of citrinin synthases and was supported by a high bootstrap value in both the phylogenetic trees. PksCT from *Monascus purpureus* is a NR-PKS containing a MeT domain downstream of ACP, the apparent result of domain arrangement [29], but consistent with the need for a methylation step in the citrinin biosynthesis [37]. Homologous of citrinin PKSs were found in other fungal species, some of them belonging to *Aspergillus* genus known to be able to produce citrinin. All of these fungal species with homologous citrinin PKSs belong to the same phylogenetic class of *Eurotiomycetes* suggesting a common ancestor in the evolution of citrinin NR-PKSs. However, the putative citrinin PKS found in *A. fumigatus* was more distant from the others, it does not contain the MeT, but an additional ACP domain, and its function has not been yet established. The second class of NR-PKSs (100% and 91% bootstrap values for KS and AT phylogenies, respectively) do not have MeT domain, suggesting that the gene encodings these PKSs diverged after the loss of the Met domain, as reported by Kroken *et al.* [29]. Among the others, this class contained the enzymes involved in the biosynthesis of aflatoxins and sterigmatocystin. Aflatoxins consist of at least 16 structurally related furanocoumarins, with AFB_1_, AFB_2_, AFG_1_ and AFG_2_ being the four most abundant and with *A. flavus* and *A. parasiticus* being the most prominent producers. Aflatoxins share an analogous biosynthetic route with sterigmatocystin, the penultimate intermediate of aflatoxin, a carcinogenic polyketide produced by several fungal species in different genera. The initial step in aflatoxin and sterigmatocystin biosynthesis is the fatty acid synthase catalyzed formation of the starter unit hexanoic acid, which is then extended to norsolorinic acid by a NR-PKS in which the two previously unrecognized SAT, and PT domains were identified [38,39,40]. Dothistromin is a mycotoxin, produced by *Mycosphaerella pini*, a fungal forest pathogen, remarkably similar in structure to versicolorin B, a precursor of both aﬂatoxin and sterigmatocystin. The PKS required for its production is unusual in that the ACP domain is present in triplicate [41]. In both the phylogenetic trees of KS and AT domain, the PKS enzymes related to these three toxins clustered together at a high bootstrap (100% and 98%, respectively). However, according to our analyses, dothistromin KS domain was more correlated to the KS domain of sterigmatocystin PKS, on the contrary AT domain was grouping with the AT domains of aflatoxin PKSs. From alignment analyses, the third ACP was hypothesized to have been acquired by unequal recombination in the fungus and then diverged into a slightly different form with less functionality or altered specificity [41]. The presence of homologous aflatoxin and sterigmatocystin PKS genes from *A. flavus* and *A. parasiticus* (Class *Eurotiomycetes*) in the genus *Mycosphaerella* belonging to the Class *Dothideomycetes* leads to the potential for horizontal transfer of genes between these disparate classes of fungi, which probably evolved on the same host/substrate. 

The group of aflatoxin PKSs contained also PKSs from nonaflatoxigenic *A. sojae* and *A. oryzae.* In *A. sojae* the PKS sequence contains a pretermination stop codon and encodes a truncated product: the KS and AT domains are present and are identical to the domains of the other producing *Aspergillus* species, whereas the TE domain is lacking. However, the lack of aflatoxin producing ability of *A. sojae* is primarily due to the truncation of the transcriptional activation of the regulatory gene *AflR* [42]. In the industrial specie *A. oryzae*, aflatoxin PKS displays a 99% similarity to PKSs of producing species; the nonaflatoxigenicity of this fungus is the result of several mutations in different genes of biosynthetic cluster [43]. Duplicate ACP domains are common in fungal PKSs like the NR-PKSs responsible of the biosynthesis of cercosporin toxin produced by *Cercospora nicotianae* and *C. coffeicola* [44], which were close to the aflatoxin PKSs group. In this study, we also considered PKSs involved in the biosynthesis of pigments (melanin precursor naphthopyrone and red pigment bikaverin) because of their importance as fungal secondary metabolites [45]; their biosynthesis PKSs, containing two ACP domains, except for *Arthroderma otae* PKS displaying only one ACP domain, were included in the same subclade of aflatoxins NR-PKSs. In the NR-PKS clade, the PKS13 involved in zearalenone biosynthesis was included, but it resulted clearly separated from the other NR-PKSs with a high bootstrap in both the domain analyzed (100% for KS and 91% for AT), evidencing a divergence in its evolutionary origin. Zearalenones are produced by several species of *Fusarium* and their biosynthesis involves two divergently transcribed PKS genes, sharing the same promoter and termed *pks13* and *pks4* (or *zea1* and *zea2*), respectively [46,47]. The second PKS (PKS4) is an HR-PKS and grouped correctly in the HR clade, which was identified by bootstrap values of 67 and 91%, in KS and AT trees, respectively. An analog system with two PKSs acting on the same growing polyketide is the T-toxin produced by *Cochliobolus heterostrophus*, in which two HR-PKS (PKS1 and PKS2) are required to synthesize the linear carbon chain [48], and both clustered in the HR-PKSs clade. However, PKS1 of *C. heterostrophus* and the homologous PKS1 of *Mycosphaerella zea-maydis*, necessary for the synthesis of structurally similar PM-toxin [49], appeared phylogenetically distinct from PKS2, which had a clearly separate placement in both the domain phylogenies with respect to other HR-PKSs (bootstrap of 67% and 91% in KS and AT domains, respectively). The two *C. heterostrophus* PKSs proteins are different partly because in PKS1 a degenerate MeT domain is present, with poorly conserved motifs that likely are non-functional, considering that T-toxin molecule is not methylated [48]. The clade resulting phylogenetically related to PKS1 T-Toxin, in KS domain, but not in AT domain, comprised the LovF of *A. terreus*; the two PKS (PksH and PksJ) involved in the biosynthesis of alternariol and the PKS involved in the biosynthesis of AF-toxin, both produced by *Alternaria alternata*; and the *Aspergillus* OTA related PKSs. In particular, the two HR-PKSs responsible of alternariol biosynthesis resulted highly different in AT domain and more closely related in KS domain. PksJ resulted phylogenetically related to *Aspergillus* OTA-PKS in both domains, while PksH is highly related to the LovF of *A. terreus* in KS domain and between the hybrid PKS-NRPS and fumonisins clades in AT domain. These results are in accordance with the evidence that PksH, having a likely regulatory role, shows an additional CMeT domain compared with PksJ and is located in a different gene cluster from PksJ [50]. In this respect, alternariol and alternariol-9-methyl ether, exhibiting cytotoxic, foetotoxic, teratogenic and suspicious mutagenic effects, are contaminants of food such as cereals, fruits and fruit juice. Instead, the lovastatins are among the most important and studied secondary metabolites produced by fungi, utilized for their pharmacological activity, and therefore, they were considered in this study. Two PKSs are involved in their biosynthesis as demonstrated in *A. terreus* [51]: the HR-PKS LovF and LovB, the latter containing a MeT domain, but not a ER domain and also possessing a C-terminal truncated C domain; it is not clear whether this domain has a function in offloading or if it is just a relict of truncation. The ER activity involved in lovastatin synthesis is provided by the additional protein LovC. Lovastatin LovF is noniterative, differently from all known fungal PKSs that are iterative; in fact, it synthesizes a diketide for which only a single elongation step is required. Each of the two PKSs synthesizes an independent PK chain, and then they are linked via an ester bond, catalyzed by LovD [52]. As shown in Figure 1, Figure 2, differently from LovF, LovB together with its putative homologues found in other fungal species, resulted scattered within the hybrid PKS-NRPS clade. This phylogenetic position, likely due to the presence of the C domain in the molecular structure of LovB, suggests a hypothetical derivation from an ancestral hybrid PKS-NRPS. 

A branch of the HR-PKS clade grouped the fumonisins PKSs with a consistent bootstrap of 100%, in both KS and AT trees. These are a mycotoxin group of great importance, as they are suspected to be carcinogenic to humans and are as consequence regulated mainly in maize-based products. To date, at least 28 fumonisins have been isolated from fungi and they can be divided into four groups A, B, C and P; in the B series, FB_1_ is the most toxic fumonisin analog [53]. The HR-PKS FUM1, containing a MeT domain, was the first characterized member of the fumonisin gene cluster [54]. The surprising discovery of putative homologues to the *F. verticillioides* fumonisin gene cluster in two different *A. niger* genomes [55,56] led to the subsequent documentation of actual FB_2_ and FB_4_ production and of a new B-series fumonisin FB_6 _in *A. niger* [57,58]. From a current screening project, it appears that fumonisins are much more common than OTA production within *A. niger* [59,60]. The grouping of both domains of fumonisin PKSs in a subclade closely related to the hybrid PKS-NRPSs, suggests a possible similar evolution history with hybrid synthetases. In fact, the biosynthesis of some mycotoxins, like fumonisins, needs the activity of both PKS and NRPS, usually for the formation of polyketide molecule linked to an amino acid residue. In some cases, the two enzymes act individually in different steps of the biosynthetic pathway and they are located adjacent in the gene cluster, alternatively the two enzymes are combined in a hybrid system, which displays the principal domains of NRPS sequential to the PKS domains in the same protein structure. Examples of these hybrid synthases are the enzymes for biosynthesis of α-cyclopiazonic acid (CPA) produced by several *Aspergillus* and *Penicillium* species. This mycotoxin is noted to be co-expressed with aflatoxin [61]. The PKS portion of the CPA enzyme catalyzes diketide formation while the NRPS portion catalyzes the condensation to tryptophan. In addition, the biosynthesis of fusarins, secondary metabolites from *Fusarium* species, their most prominent member being fusarin C, requests a PKS-NRPS hybrid, the first hybrid identified in filamentous fungi [62], for the formation of a heptaketide linked to homoserine. The PKS-NRPS PsoA in *A. fumigatus* is involved in the biosynthesis of pseurotin A [63]. Cytochalasins comprise a miscellaneous group of fungal polyketides with an impressing variety of biological activities; beside cytotoxic also antibiotic, anti-inflammatory, antitumoral and antiviral activities. Among them, chaetoglobosin A biosynthesis was investigated in *P. expansum* [64] leading to the identification of a PKS-NRPS (CheA) for the production of a trimethylated nonaketide fused to a tryptophan moiety. Unlike chaetoglobosin A, cytochalasin E, produced by *A. clavatus,* is derived from a shorter polyketide chain and a different amino acid building block (phenylalanine instead of tryptophan). According to both KS and AT phylogenetic analyses, the PKS-NRPS (CcsA) involved in its biosynthesis [65] seemed to be phylogenetically closer to EqiS than to CheA, despite the significant structural difference. EqiS is the PKS-NRPS responsible of the biosynthesis of equisetin in *F. heterosporum* [66]; it forms a unique fungal natural product backbone: a lovastatin-like decalin of polyketide origin fused to serine. In fact, it resulted close to one of the PKSs involved in lovastatin biosynthesis (LovB). 

The clade of PR-PKSs, that comprises 6-methyl salicylic acid synthases, was highly supported by a bootstrap of 100% in both KS and AT trees. The formation of the polyketide methylsalicylic acid is the primary building block of the biosynthetic pathway of patulin, a mycotoxin produced by a number of different *Penicillium* species [67]. In the phylogenetic analyses conducted by Kroken *et al.* [29], the fungal clade of 6-MSAS was nested within the large clade comprising all bacterial type I PKSs, suggesting an origin of these fungal PKS from bacteria by one or more HGT events. From our analyses, the PKS involved in OTA biosynthesis in *P. nordicum*, whose deposited partial sequence only displays KS and AT domains, was also included in the 6-MSAS clade. Ochratoxins are nephrotoxic and carcinogenic mycotoxins produced by a number of *Aspergillus* and *Penicillium* species [11]. The molecular structure of OTA suggests a biosynthetic pathway including, among other enzymatic steps, a PKS for the synthesis of the pentaketide dihydroisocoumarin and a NRPS to catalyze the ligation of phenylalanine with the PK. OTA PKS genes have been partially characterized in *A. ochraceus* [68], *A. westerdijkiae* [69] and *P. nordicum* [70]. In the recently sequenced genome of *A. carbonarius* (http://jgi.doe.gov/carbonarius/) a putative OTA cluster was identified containing a PKS, which shows a high similarity to the predicted OTA cluster *pks* gene of the producing *A. niger* strain CBS 513.88 [56]. The PKS related to OTA biosynthesis from different fungal species were distributed in different classes in the KS and AT phylogenetic trees. Putative OTA PKSs identified in the genomic sequences of *A. carbonarius* and *A. niger* and the OTA PKS of *A. ochraceus* were grouped together in the HR-PKS. For *A. ochraceus*, the sequence of a previously deposited partial PKS, overlapping the AT domain of the gene characterized by O’Callaghan *et al.* [68], was considered for KS analysis. In *A. westerdijkiae*, a new species of fungus that was recently dismembered from *Aspergillus ochraceus* taxon, a different PKS required for biosynthesis of OTA was identified. Only its AT and KS domains were cloned and sequenced, and they resulted in being phylogenetically related to LovB PKSs in the hybrid synthetase group. Differently, the OTA PKS of *P. nordicum* clustered with 6-MSAS, as reported above. In a previous study, the partial sequence containing only KS and AT domains of an additional PKS (accession no. CAQ16344) had been identified in *A. carbonarius*, whose expression pattern showed a correlation to OTA production under permissive conditions [71] and that clustered close to the hybrid PKS-NRPS group. 

### 2.2. Analysis of A and C Domains of NRPSs

**Figure 3:**
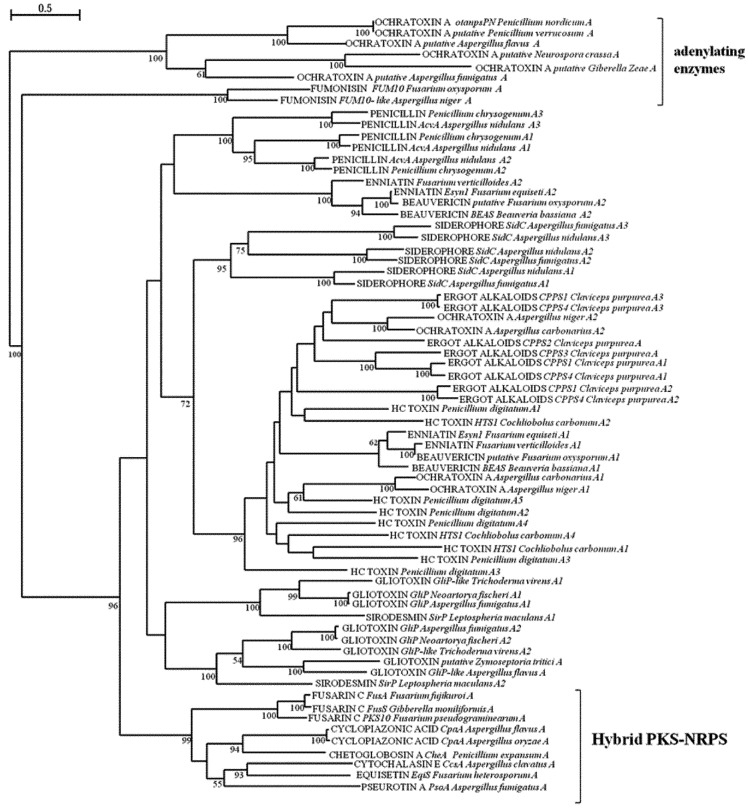
ML phylogenetic tree of adenylation (A) domains from NRPSs and hybrid PKS-NRPS based on RAxML algorithm. Numbers at nodes indicate bootstrap support, reported when ≥50% for each clade performed with 1,000 replications. A1-An indicates the number of A domains in structures of NRPSs and hybrid PKS-NRPS.

**Figure 4:**
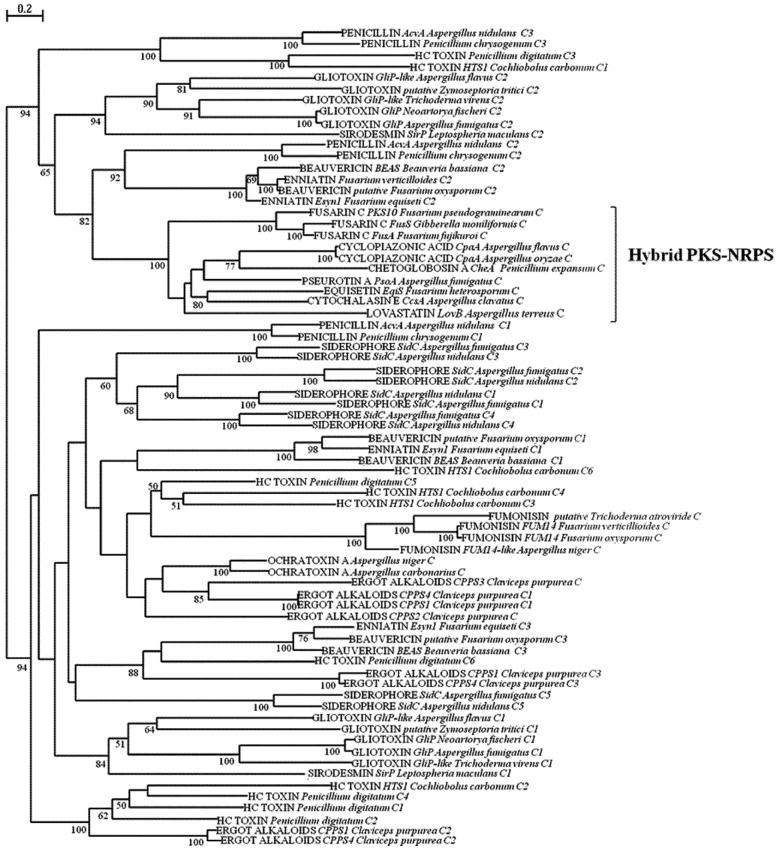
ML phylogenetic tree of condensation (C) domains from NRPSs and hybrid PKS-NRPS based on RAxML algorithm. Numbers at nodes indicate bootstrap support, reported when ≥50% for each clade performed with 1,000 replications. C1-Cn indicates the number of C domains present in structure of NRPSs and hybrid PKS-NRPSs.

The second group of fungal secondary metabolites considered in this study is constituted by peptidic products generated by NRPSs. Fungal NRPs have been found to have a wide range of biological activities. In addition to toxins and mycotoxins involved in plant and animal pathogenesis, they also comprise antibiotics, immunosuppressant and anticancer agents, used as pharmaceuticals and compounds involved in different fungal activities, such as nutrition, growth and development, reproduction, pathogenicity and stress management. The varied modular structure of NRPSs entails complex evolutionary mechanisms at the basis of their origin, such as duplication and loss of modules or domains, gene fusion, recombination or conversion of modules or domains in the same NRPS or among different NRPSs. In this study, we performed the phylogenetic analysis of A and C domains of NRPSs and hybrid PKS-NRPSs involved in the biosynthesis of mycotoxins (Figure 3, Figure 4), with the intent to deduce possible evolutionary relationships among the biosynthesis mechanisms of different mycotoxins in different fungal species. Differently from the KS and AT phylogenetic analyses of PKSs, there was not a clear clusterization according to the structural diversity of NRPSs. For example, it was not possible a separation between mono/bimodular and multimodular enzymes as delineated in the work of Bushley and Turgeon [32], in which the A domains of a much greater number of NRPSs of different origin and function were considered and multimodular NRPSs were suggested to be of more recent origin and with a less stable domain architecture. However, a concordance was found in the well-defined separation of a clade including NRPS-like enzymes consisting of a truncated module with A and PCP domains, that Bushley and Turgeon [32] defined as adenylating enzymes and selected as potential outgroup. According to our analyses, also the A domains of FUM10 clustered separately. In fumonisin biosynthetic cluster FUM7, FUM10 and FUM 14, seem to represent a NRPS-like complex, where FUM10 is analogous to the A domain, FUM14 contains the PCP and C domains, and FUM7 may be regarded as a reductase domain [72,73]. Only the catalytic action of esterification by FUM14 has been demonstrated *in vitro*. FUM14 represents the first example of an NRPS-like enzyme catalyzing a C-O bond formation instead of the typical C-N bond [74]. In the genome of *A. niger,* a homolog of FUM 10 has been also found in relation to its capacity of fumonisin production. Regarding the phylogeny of FUM 14, its C domain grouped in a subclade together with domains from complete mono, bi and multimodular NRPSs. The evolutionary origin of the four domain NRPS complex of fumonisin biosynthesis is unknown, the phylogenetic position of FUM14 could suggest the origin from an initially complete ancestral NRPS as they clusterized all together with a bootstrap of 100% for their condensation domain. 

The clustering of NRPs portion of hybrid PKS-NRPSs in a subclade was supported by bootstrap value of 99 and 100% in A and C phylogenetic trees, respectively. However, the PKS-NRPSs sub-clade in A phylogenetic tree constituted a distinct clade clearly separated from other NRPS A domains, with respect to C phylogenetic tree, where PKS-NRPSs, although belonging to the same sub-clade, clusterized with other non-hybrid NRPSs C domain. This result sustains the hypothesis of their common origin, as evidenced also by the phylogenetic analysis of their PKS domains. In this group also the C domain of PKS LovB of lovastatin biosynthesis was present, confirming the result obtained with the analyses of its AT and KS domains and suggesting the possibility of a common ancestor with hybrid synthases, which could have lost the A and PCP domains during evolution. Gliotoxin is the main member of the epipolythiodioxopiperazine (ETP) class of natural products characterized by a disulfide bridged cyclic dipeptide. A two module NRPS, GliP, was shown to be involved in the biosynthesis in *A. fumigatus* [75]. Another member of the same class of products is sirodesmin, a phytotoxin from *Leptosphaeria maculans* [76]. The two toxins share the same biosynthetic principle: two amino acids (for gliotoxin l-serine and l-phenylalanine; for sirodesmin l-serine and l-tyrosine) are cyclized by formation of two peptide bonds. The A and C domains of NRPSs for gliotoxin and sirodesmin grouped together; with the two A domains from these NRPSs resulting more related between them than the two C domains, which appeared separated in the two major clades arose from the C domain analysis. It has been observed that A domains within a single biosynthetic pathway are often very similar to each other in spite of activating different amino acids [77]; in fact, A_1 _and A_2 _of gliotoxin NRPSs belonged to the same clade. In this respect, putative gliotoxin NRPSs in *Zymoseptoria tritici* and *A. flavus* present a truncated structure with one only A domain, which corresponded to the A_2_ domain of other gliotoxin NRPSs as depicted in Figure 3. The NRPSs involved in enniatin and beauvericin synthesis display an unusual bimodular layout with two A domains and three C domains, two of which on either end forming a cyclization cavity. Enniatins and beauvericin are cyclohexadepsipeptides produced by various *Fusarium* species and *Beauveria bassiana* and C_1_ and C_3_ domains contribute to the cyclization of the molecule [78]. From our analysis, these two domains were closer between them than to the C_2_ domain, but all the three domains resulted phylogenetically unrelated. However, C_1_ domain resulted more related to the last domain of a multimodular NRPS (HTS1) involved in the biosynthesis of HC toxin, a cyclic tetrapeptide that is a host selective fungal toxin produced by race 1 isolates of *Cochliobolus carbonum* [79]. HTS1 is an example of lineage specific synthetases, which tend to have more specialized, niche specific functions. In fact, they appear to be unique even to one race or pathotype within a single species. In genome of *Penicillium digitatum* a putative homologous was found, which displays a similar domain structure, even though with a greater number of domains apparently due to duplication events. All the A domains of these NRPSs grouped in a subclade comprising the two A domains of OTA NRPS of *A. carbonarius* and *A. niger*, the A domains of NRPSs involved in ergot alkaloids biosynthesis and the first A domain of enniatin/beauvericin NRPSs, supported by a significant bootstrap value (96%). Differently, the C domains of HC toxins were distributed along the different subclades identified by the phylogenetic analysis. The presence of homologous domains arranged in different combinations in NRPSs of various functions and fungal origin is likely due to events of recombination in the evolution of multimodular NRPSs. 

As written above, in OTA biosynthesis the chemical bound of phenylalanine to the polyketide dihydroisocoumarin structure is catalyzed by a NRPS. In *A. carbonarius* a monomodular, NRPS was demonstrated to be involved in the biosynthesis. It is constituted by a complete domain likely functional to the bound of phenylalanine and an additional A domain at the end whose role is still not clear. In addition, experimental data provided the first evidence that this enzymatic step precedes the chlorination step to form OTA, contributing to its toxicity, and that OTα is a product of hydrolysis of OTA, clarifying the order of reaction in OTA biosynthetic pathway, until then not completely elucidated [80]. The second additional A domains of the two NRPS involved in OTA in *Aspergillus* grouped together with the A_3_ domains of the two multimodular NRPSs found in the ergot alkaloids biosynthesis cluster. In *P. nordicum* a NRPS-like protein was reported to be responsible of OTA biosynthesis [70]; it is formed by an incomplete module lacking the C domain. Its A domain was distant from A domains of *Aspergillus* OTA NRPSs and grouped in the clade separately from other NRPSs (100% bootstrap), together with other putative OTA NRPS, reported as outgroup by Bushley and Turgeon [32] as adenylating enzymes; the A domains of fumonisin FUM 10 NRPS resulted an additionally outgroup in our analysis at a bootstrap of 100%. The presence of a system consisting of different NRPSs as for fumonisin biosynthesis has been reported for the biosynthesis of ergot alkaloids in *Claviceps purpurea*, as mentioned above [81,82]. The core NRPS CPPS1 exhibits three modules for the extension of d-lysergic with the amino acids alanine, phenylalanine and proline. However, CPPS1 is lacking the first C domain, which is provided *in trans* by CPPS2. Of the two further NRPSs, CPPS3 is monomodular and CPPS4 is a copy of CPPS1 with high similarity and certainly arose by a gene duplication event. The current biosynthesis model explains the coexistence of CPPS1 and CPPS4 as a basis for the concurrent biosynthesis of ergotamine and ergocryptine, which shares the d-lysergic acid starter, but differs in the amino acid extension. NRPSs synthesizing ergot alkaloids were hypothesized to have originally evolved to function in animal pathogenesis [32]. A similar phylogenetic correlation found between all the A domains of these NRPSs and the second A domains of OTA NRPS of *A. carbonarius* and *A. niger*, was also evident between the C domain of the same OTA NRPSs and the C domains of monomodular CPPS2 and CPPS3 and the first C domains of trimodular CPPS1 and CPPS4, suggesting the sharing of a common origin. In the light of this, it is also interesting to observe that the A_1_ domain of *Aspergillus* OTA NRPS grouped with some A domains of HC toxin and that the C_2_ domain of trimodular CPPSs grouped with C domains of HC toxin NRPS. In our analyses, we also included some non-toxic metabolites of peptidic origin, like penicillins, important for their beneficial effects and their pharmaceutical utilization. The first step in penicillin biosynthesis is carried out by d-(l-α-aminoadipyl)-l-cysteinyl-d-valine (ACV) synthetase, which forms peptide bonds between the three precursor amino acids [83]. This group of trimodular NRPSs has been postulated to be horizontally transferred from bacteria to fungi [84,85]. In our analyses, whereas the three A domains grouped together, the three C domains were distributed in different subclades, comprising also C domains from NRPSs responsible of toxin production. Regarding the analysis of fungal NRPSs involved in other aspects of fungal metabolism, we considered the enzymes which biosynthesize intracellular siderophores (SID). Siderophores play very diverse roles: their primary function is the acquisition of iron under iron-limited conditions and its storage; some siderophores are also required for resistance to oxidative stress, asexual/sexual development or virulence [86]. Here we considered Sid C from *Aspergillus* with three complete modules plus two additional peptidyl carrier domain/condensation domain-units. Siderophores grouped as a subfamily in the A domain phylogenesis of Bushley and Turgeon [32]; in our study all the A domains clustered together in a distinct subclade (95% bootstrap), like so the first four C domains (bootstrap 60%), while the last C_5_ domain clustered separately from the other, together with the last C domain of enniatin/beauvericin NRPSs and the last domain of trimodular ergot alkaloids NRPSs, although this subclade was not supported by a valid bootstrap value.

## 3. Experimental Section

### 3.1. Identification of PKS and NRPS Involved in Mycotoxin Biosynthesis

The amino acid (aa) sequences data set of the selected PKSs, NRPSs and PKS-NRPS hybrids, known to be involved in mycotoxin biosynthesis, were derived from the online database of the NCBI (http://www.ncbi.nlm.nih.gov/), Broad Institute (http://www.broadinstitute.org/) and JGI (http://genome.jgi.doe.gov/). The information about the real or putative involvement of the selected protein in the relevant mycotoxin biosynthesis was recovered from the published literature. Some non-toxic metabolites of polyketide and peptide origin were also included in the phylogenic analyses, like pigments (CAB92399, BAB88689, XP_002842704, AAC39471, Q03149), penicillins (P26046, CBF84349) and fungal NRPSs involved in the biosynthesis of intracellular siderophores (SidC) (EAL91050, CBF89140). 

### 3.2. Identification of Enzymatic Domains

The accepted common strategy for the identification of a specific type of domain is based on the use of the Profile Hidden Markov Models (pHMMs); these models are suited for the identification of motifs interrupted by segment of variable length and to characterize position-specific sequence similarities among family of proteins. In fact, collection of pHMMs for the various domains and domains families are available on database like Pfam [87], HMMER and TIGRFAMs [88] and were queried through SMART (http://smart.embl-heidelberg.de/) and CDD (http://www.ncbi.nlm.nih.gov/Structure/cdd/cdd.shtml) bioinformatic software. Then using this bioinformatic approach the different KS and AT domains from PKSs and A and C domains from NRPSs were identified in the corresponding aminoacidic sequences. The complete list of the accession number of the downloaded sequences and of the annotated domain are summarized in Table 1. Because of mono/bimodular and multimodular structure of NRPSs, the multiple A and C domains were kept separately and coded numerically according to their iterative succession (e.g., A_1_–A_n_ and C_1_–C_n_). A complete PKS-NRPS hybrid protein was identified on the basis of the occurrence of one complete module with C and A domains in NRPS portion and the KS and AT domains in PKS portion. 

### 3.3. Multiple Sequence Alignment and Phylogenetic Analysis

The reconstruction of a good and reliable phylogenetic tree is crucially dependent on the right protein alignment and the best substitution model used in the phylogenetic analysis of the data. All the aa sequences domain KS and AT for PKS and A and C for NRPS were aligned with MUSCLE [89]. MEGA5 software [90] was used to identify an appropriate protein substitution matrix to avoid inaccurate phylogenies. The Whelan and Goldman (WAG) model had the best likelihood for all the criteria. The genealogy of 58 KS and 58 AT domains for PKS and the genealogy of 68 A and 73 C domains for NRPS on the basis of the obtained alignment was primarily inferred by neighbor joining and maximum parsimony analysis using the *MEGA* version 5. The Maximum Parsimony (MP) trees were obtained using the Close-Neighbor-Interchange algorithm with search level 3 in which the initial trees were obtained with the random addition of sequences (10 replicates). Subsequently, after the preliminary phylogenetic results and the removal from the analysis of the not well supported sequences the Maximum Likelihood (ML) method was used for phylogeny construction using three different algorithms: RaxML-HPC [91] at Phylobench.vital-it (http://phylobench.vital-it.ch/raxml-bb/); MEGA5 [90]; PhyML 3.0 [92] at Phylogeny.fr (http://www.phylogeny.fr/). Bootstrapping was performed to assess the robustness of the phylogeny. The most robust approach to infer phylogenetic relationship among the four domains studied was ML analysis by using the RAxML algorithm, setting the bootstrap analysis to 1000 runs with the best scoring ML tree used for phylogenetic analysis, the number of characters were 525 for KS, 435 for AT, 542 for A and 412 for C domains, respectively. The final ML optimization Likelihood values were -24909.988807, -25353.725213, -44270.460459, -41195.965573 for KS, AT, A and C domains, respectively. Trees were drawn using NJplot [93] and manually edited.

## 4. Conclusion

The great diversity in the chemical structure of fungal mycotoxins, as well as the fact that a particular toxin may be produced by different fungal species, point out the complexity of molecular evolution that fungi have exploited for the production of these dangerous metabolites. The backbones of numerous mycotoxins are synthesized by PKSs or NRPSs and the biosynthesis of some other mycotoxins requires the activity of both the enzymes. In our study, we have performed the phylogenetic analyses of the main domains of these megasynthases involved in the biosynthesis of principal mycotoxins. Regarding PKSs, results obtained from KS and AT domains phylogenies agreed on the identification of two clades corresponding to the two classes of not-reducing and reducing PKSs. This latter clade was then divided in the two subclades of partially and highly reducing PKSs. Hybrid PKS-NRPSs were included in the major clade of HR-PKSs, supporting the hypothesis of a common ancestor. Hybrid synthetases grouped in defined subclades also in the phylogenetic trees of A and C domains of the corresponding NRPS portions, with the A domains being more supported by bootstrap values. The A domains tree allowed the differentiation of complete NRPS not only from hybrid enzymes, but also from NRPS-like proteins involved in mycotoxin biosynthesis and that are characterized by an incomplete modular structure. This group included the OTA NRPS of *P. nordicum*, which presents in its structure the A and PCP domains and lacks the C domain, and the other putative OTA, like proteins selected in the phylogenetic study of Bushley and Turgeon [32] as adenylating enzymes. We also found FUM10 of fumonisin biosynthesis cluster separated from the regular NRPSs and from OTA-like proteins, due to the presence in their structure of only a A domain. For most mycotoxins, the corresponding PKSs and NRPSs from different fungal species grouped together, suggesting the hypothesis of events of HGT between fungi. Generally, such events were reported to involve the transfer of a single or a few genes between very distantly related organism belonging to different kingdoms [94,95], whereas fewer findings of these events between eukaryotes were described. Actually, the clustering of biosynthetic genes for secondary metabolites in fungi could made possible the transfer of a whole pathway in a single event. Recently, the horizontal transfer of sterigmatocystin gene cluster in *Podospora asperina* from *Aspergillus*, two fungi belonging to different taxonomic classes, was reported [96], suggesting that HGT events between fungi could have contributed to fungal metabolic diversity. 

A different event could be hypothesized for the evolution of genes involved in OTA biosynthesis in *Aspergillus* and *Penicillium* genera. From our analyses, PKSs and NRPSs responsible for OTA production in *Aspergilli* appeared phylogenetically very distant from the two enzymes in *P. nordicum*. PKSs from *A. carbonarius*, *A. niger* and *A. ochraceus/westerdijkiae* were included in the HR-PKS group, even though a complete protein structure was available only for *A. carbonarius* and *A. niger* PKSs. The analysis of KS and AT domains, unique domains available for OTA PKS in *P. nordicum*, permitted us to classify it among PR-PKSs, close to 6-methyl salicylic acid synthases. A similar phylogenetic distance was observed for OTA NRPSs, which were structurally diverse, with the NRPS from *P. nordicum* lacking a C domain and grouping in a clade separate from regular NRPSs, differently from the enzymes from *Aspergilli*, which present a complete module plus an additional A domain. This situation could be explained by a process of convergent evolution of non-homologous gene clusters toward OTA biosynthesis rather than a separation from a common ancestor [97]. The particularity of high phylogenetic divergence of OTA PKS and NRPS conserved domain among *Penicillium* and *Aspergillus* genera point out the fact that retrieving information or activity on unknown proteins by similarity or homology to known amino-acidic sequences could lead to erroneous identification (as the case of the putative OTA NRPSs identified in *Neurospora*, *Aspergillus* and *Fusarium* genera by Bushley and Turgeon [32]). In addition, the C and the second A domains of *Aspergillus* OTA NRPSs revealed an interesting phylogenetic proximity to the domains of the NRPSs involved in the biosynthesis of ergot alkaloids, suggesting especially for the C domain a possible common origin for the biosynthetic genes of these two toxins.

Our analyses clearly evidenced and confirmed previous hypothesis on the complex evolutionary history through which these multimodular enzymes had developed and diversified, including gene duplications, modular rearrangements, potential gene loss and horizontal transfer. A better understanding of these evolution events could help to further clarify the mechanisms underlying biosynthesis of mycotoxins, to link specific metabolites with the corresponding enzymes and to discover new toxic metabolites and new toxigenic fungal species. Finally, the phylogenetic analysis of KS and AT domains evidenced with some minor exceptions the co-evolution of these two important conserved domains in PKS enzymes; unlike the A and C domains of multimodular NRPSs, which showed a divergent phylogenetic topology. In addition, especially in C domain tree, the different modules of the same NRPS resulted often phylogenetically unrelated, apart for the four C domains of siderophore, evidencing that their origin is not from duplicate event or co-evolution process, but probably is more related to their functional substrate specificity, as demonstrated by Rausch *et al.* [98]. Finally, our findings showed a more complex evolutionary situation for the NRPS enzymes responsible of mycotoxin synthetase than for the PKS ones. 
